# Transcriptome Analysis of the Molecular Patterns of Pear Plants Infected by Two *Colletotrichum fructicola* Pathogenic Strains Causing Contrasting Sets of Leaf Symptoms

**DOI:** 10.3389/fpls.2022.761133

**Published:** 2022-02-16

**Authors:** Min Fu, Qing Bai, Hui Zhang, Yashuang Guo, Yuhong Peng, Pengfei Zhang, Liang Shen, Ni Hong, Wenxing Xu, Guoping Wang

**Affiliations:** ^1^Hubei Hongshan Laboratory, Wuhan, China; ^2^State Key Laboratory of Agricultural Microbiology, Wuhan, China; ^3^Key Laboratory of Horticultural Crop (Fruit Trees) Biology and Germplasm Creation of the Ministry of Agriculture, Wuhan, China; ^4^Hubei Key Laboratory of Plant Pathology, Wuhan, China; ^5^College of Plant Science and Technology, Huazhong Agricultural University, Wuhan, China

**Keywords:** pear, *Colletotrichum fructicola*, early defoliation, transcriptome sequencing, phytohormone

## Abstract

*Colletotrichum fructicola* infects pear leaves, resulting in two major symptoms: tiny black spots (TS) followed by severe early defoliation and big necrotic lesions (BnL) without apparent damage depending on the pathotypes. How the same fungal species causes different symptoms remains unclear. To understand the molecular mechanism underlying the resulting diseases and the diverse symptoms, two *C. fructicola* pathogenetic strains (PAFQ31 and PAFQ32 responsible for TS and BnL symptoms, respectively) were inoculated on *Pyrus pyrifolia* leaves and subjected to transcriptome sequencing at the quiescent stage (QS) and necrotrophic stage (NS), respectively. *In planta*, the genes involved in the salicylic acid (SA) signaling pathway were upregulated at the NS caused by the infection of each strain. In contrast, the ethylene (ET), abscisic acid (ABA), and jasmonic acid (JA) signaling pathways were specifically related to the TS symptoms caused by the infection of strain PAFQ31, corresponding to the yellowish and early defoliation symptoms triggered by the strain infection. Correspondingly, SA was accumulated in similar levels in the leaves infected by each strain at NS, but JA was significantly higher in the PAFQ31-infected as measured using high-performance liquid chromatography. Weighted gene co-expression network analysis also reveals specific genes, pathways, phytohormones, and transcription factors (TFs) associated with the PAFQ31-associated early defoliation. Taken together, these data suggest that specific metabolic pathways were regulated in *P. pyrifolia* in response to the infection of two *C. fructicola* pathotypes resulting in the diverse symptoms: JA, ET, and ABA accumulated in the PAFQ31-infected leaves, which negatively affected the chlorophyll metabolism and photosynthesis pathways while positively affecting the expression of senescence-associated TFs and genes, resulted in leaf yellowing and defoliation; whereas SA inhibited JA-induced gene expression in the PAFQ32-infected leaves, which led to hypersensitive response-like reaction and BnL symptoms.

## Introduction

Pear fruits have rich nutritional value and other beneficial food values and are ranked as the third most important temperate fruit crop after grapes and apples and are widely cultivated in six continents with major production in China, United States, Italy, Argentina, and Spain, containing five commercially cultivated species of *Pyrus bretschneideri*, *P. communis*, *P. pyrifolia*, *P. sinkiangensis*, and *P. ussuriensis* (Wu et al., 2013; Wang W. H. et al., 2019). Pear anthracnose caused by *Colletotrichum* spp., is one of the destructive diseases in major pear-cultivation areas of China (Fu et al., 2019). It occurs in pear’s growth and fruit maturation periods and mainly damages leaves and fruits, resulting in great economic losses (Wu et al., 2010; Zhang et al., 2015). Of these species, *Colletotrichum fructicola* has been characterized as the most predominant species related to the pear anthracnose in China (Fu et al., 2019). Usually, *C. fructicola* induce big sunken rot lesions (BrL) on the fruits and big necrotic lesions (BnL) on the leaves of *P. pyrifolia*, *P. bretschneideri*, and *P. communis* (Li et al., 2013; Jiang et al., 2014; Fu et al., 2019). In recent years, *C. fructicola* has been identified as responsible for other kinds of symptoms characterized by tiny black spots (TS) (diam. < 1 mm) on *P. pyrifolia* leaves, followed by leaf yellowing, and finally, severe early defoliation, resulting in big losses up to zero yield in recent years (Huang et al., 2015; Zhang et al., 2015). We further indicated that TS and BrL/BnL symptoms were induced by two different pathogenetic strains (two pathotypes) of *C. fructicola*, respectively (Fu et al., 2019), and the latter does not cause any further obvious damages to the infected leaves except for the necrotic lesions. The molecular mechanisms underlining the two symptoms induced by both strains remain undetermined. Since most *Colletotrichum* species only cause BrL/BnL symptoms on infected crops (Cannon et al., 2012; Huang et al., 2013; Liu et al., 2015; Munir et al., 2016), it is imperative to understand how *C. fructicola* induces the TS symptoms and early defoliation besides the BrL/BnL on *Pyrus* species.

To combat pathogen infection, host plants have evolved two sophisticated defense mechanisms: pathogen-associated molecular patterns (PAMPs)-triggered immunity (PTI) and effector-triggered immunity (ETI) (Jones and Dangl, 2006), which trigger some key signaling modules (Torres et al., 2006; Zhou and Zhang, 2020), e.g., plant hormones in response to fungal infection (Pieterse et al., 2009, 2012). Of these plant hormones, salicylic acid (SA) and jasmonic acid (JA) are considered as the backbone of the plant immune signaling network and usually play different roles in response to diverse fungal infections (Bari and Jones, 2009; Pieterse et al., 2012; Ding and Ding, 2020). Other hormones, e.g., auxins, abscisic acid (ABA), ethylene (ET), gibberellins (GAs), and cytokinins (CKs), are also involved in this process in synergistic or antagonistic roles (Choi et al., 2010; Cao et al., 2011; Pieterse et al., 2012; Zhao et al., 2019) which can optimize the defense systems in response to different pathogens or infection patterns of the same pathogen by interactions or cross talk (Pieterse et al., 2012). Besides, reactive oxygen species (ROS) accumulate (Mammarella et al., 2015; Sorokan et al., 2018) and lead to an oxidative burst that induces a hypersensitive response (HR) to limit pathogen invasion, as shown by the host resistance to *Colletotrichum* spp. (O’Connell et al., 2004; Wang et al., 2018). Multiple evidence suggests that phytohormones, ROS or peroxidases (PODs) (regulating the generation and utilization of ROS), were also associated with plant senescence (leaf yellowing and defoliation, fruit abscission, etc.) (Roitto et al., 2003; Zhang and Xing, 2008; Yang et al., 2015; Woo et al., 2019; Zhao et al., 2019). How a pear plant responds to the infection of *C. fructicola* strains resulting in diverse symptoms and early defoliation remains undetermined.

*Colletotrichum* species infect plants not only with an intracellular hemibiotrophic strategy (most common) but also in a subcuticular intramural necrotrophic manner (O’Connell et al., 2012; Gan et al., 2013; De Silva et al., 2017), and some species (e.g., *C. acutatum*, *C. fructicola*, *C. gloeosporioides*, and *C. truncatum*) can employ both strategies (Perfect et al., 1999; Arroyo et al., 2005; Ranathunge et al., 2012; Shang et al., 2020). For the intracellular hemibiotrophic species, the early infection stage is the symptomless biotrophic stage accompanied by a switch to the necrotrophic stage (NS) (O’Connell et al., 2012; Gan et al., 2013). In the biotrophic infection stage, *Colletotrichum* spp., form bulbous biotrophic hyphae enveloped by an intact host plasma membrane, absorb nutrients inside the living epidermal cells without causing apparent symptoms, i.e., a quiescent stage (QS), which is different in the time duration depending on species, host development stage, and environmental condition (Peres et al., 2005; Ranathunge et al., 2012; Alkan et al., 2015), and enter an NS when developing thin secondary hyphae to destroy host tissues (O’Connell et al., 2012; Alkan et al., 2015). For subcuticular intramural necrotrophic pathogen, *Colletotrichum* spp., grow under the cuticle within the cell wall around the epidermis and shortly form thin secondary hyphae to destruct the colonized host tissues (Perfect et al., 1999). Accompanied with the unique stage-specific lifestyles, the specific host defense responses have been illustrated by simultaneous transcriptome analyses after infection by *C. gloeosporioides* and *C. fructicola* (Alkan et al., 2015; Zhang et al., 2018). At the early stage of infection, PTI and ETI were activated in host plants against the colonization of *Colletotrichum* spp., followed by a variety of defense responses, such as the changes of intracellular Ca^2+^ concentration, activation of the mitogen-activated protein kinases (MAPKs) and plant hormones signal transduction pathway, production of ROS, and generation of host programmed cell death (PCD) (Pieterse et al., 2012; Wang et al., 2018; Zhou and Zhang, 2020). However, the molecular mechanisms related to diverse symptoms triggered by the same *Colletotrichum* species are far less documented.

Here, we conducted the transcriptome analyses of sandy pear (*P. pyrifolia*) leaves at different infection stages (QS and NS) after infection by both pathotypes of *C. fructicola*. The results revealed specific host defense strategies in response to the infection of both *C. fructicola* pathotypes. These data also highlight the importance of specific genes, pathways, phytohormones, and transcription factors (TFs) in *Pyrus* spp., in regulating the different symptoms.

## Materials and Methods

### Fungal Isolates, Plant Material, and Leaf Inoculation

*Colletotrichum fructicola* strains, PAFQ31 and PAFQ32, were obtained from pear leaves (*P. pyrifolia* cv. Cuiguan, susceptible to pear anthracnose) that caused TS and BnL symptoms on pear, respectively (Fu et al., 2019). Plant tissue-cultured materials from *P. pyrifolia* cv. Cuiguan (3 years old) were grown in the greenhouse of the College of Plant Science and Technology of Huazhong Agricultural University. Pathogenicity analysis was performed on the attached leaves of *P. pyrifolia* cv. Cuiguan. The inoculation protocols have been previously described (Zhang et al., 2015). Briefly, the attached pear leaves (approximately 4-week-old) were selected for uniform size, color, and an absence of visual defects. Each leaf was surface-sterilized with 75% ethanol, washed with sterile water, air-dried, and inoculated with 40 μL of conidial suspensions (1 × 10^6^ conidia/mL) of *C. fructicola* by spraying the conidial suspensions with a handheld sprayer over the leaves. Control leaves were inoculated in parallel with sterile water. Five leaves were inoculated per biological replicate with three biological replicates per treatment. The inoculated leaves were subsequently incubated at 25°C, about 85% relative humidity (for 1 week) with a 12/12 h light/dark photoperiod for disease development.

For the RNA-sequencing (RNA-Seq) experiments, the samples of pear leaves infected with PAFQ31 and PAFQ32 were collected at the quiescent stage (QS) and necrotrophic stage (NS), respectively, and frozen in liquid nitrogen. Mock inoculation with sterile water at QS was chosen as the pear leaf control. Three independent biological replicates were sequenced for each treatment.

### RNA Extraction, Complementary DNA Library Construction, and Sequencing

The total RNA was extracted from leaf tissues using PureLink™ Plant RNA Reagent (Cat# 12322012) according to the manufacturer’s instructions (Invitrogen, Carlsbad, CA, United States) and genomic DNA was removed using DNase I (Takara, Shiga, Japan). The RNA quality was determined by a 1% denaturing agarose gel and a NanoDrop 2000 system (Thermo Scientific, Wilmington, DE, United States). RNA integrity was assessed using the RNA Nano 6000 Assay Kit and the Bioanalyzer 2100 system (Agilent Technologies, DE, United States). RNA-Seq transcriptome libraries were prepared following the TruSeq™ RNA sample preparation Kit from Illumina (San Diego, CA, United States) using 1 μg of the total RNA. Library preparation and sequencing were performed by Majorbio Bio-pharm Technology Co., Ltd (Shanghai, China). The libraries were generated using NEB Next^®^ Ultra™ RNA Library Prep Kit for Illumina^®^ (NEB, MA, United States) and were sequenced using an Illumina HiSeq X Ten platform in 2 × 150 bp pair-end sequencing mode. The RNA-Seq raw data have been deposited in the NCBI Sequencing Read Archive database under the project accession number PRJNA698408. The raw paired-end reads were trimmed, and the quality was controlled by using SeqPrep^1^ and Sickle^2^ with default parameters. Then clean reads were aligned to the reference genomes of the pear (*P. bretschneideri* cv. Suli)^3^ with orientation mode using HISAT2 (Kim et al., 2019) since the *P. pyrifolia* genome has not been released when we conducted this research.

### Differential Expression Genes Analyses and Functional Enrichment

To identify DEGs between two different samples, the gene expression levels were calculated according to the fragments per kilobase of transcript sequence per millions of base pairs sequenced (FPKM) method. FeatureCounts^4^ was used to quantify gene abundances. DESeq2^5^ (Love et al., 2014) was utilized for identifying DEGs. A significance analysis (| log_2_Fold Change| > 1 and *P*-value <0.05) of the results was used to identify genes that are strongly upregulated or downregulated by *C. fructicola* infection using unlogged data. Fold-changes were calculated with average transcript levels compared to control values that were, in turn, log_2_-transformed and calculated for Spearman correlation coefficients between treatments. Heatmap analyses were performed using OmicShare tools^6^. Functional-enrichment analyses including Gene Ontology (GO) and Kyoto Encyclopedia of Genes and Genomes (KEGG) enrichment analyses were performed to identify which DEGs were significantly enriched in GO terms and metabolic pathways at a corrected *P*-value ≤ 0.05 compared with the whole-transcriptome background. GO functional enrichment and KEGG pathway analyses were carried out by Goatools^7^ and KOBAS^8^. The analyses were based on the online platform of Majorbio Cloud Platform^9^.

### Gene Co-expression Network Analysis and Protein-Protein Interaction Network Construction

Gene co-expression network analysis was performed using the weighted gene co-expression network analysis (WGCNA) package^10^ based online platform of Majorbio Cloud Platform. Gene cluster dendrogram was constructed with colors based on the correlations between the expression levels of genes and used to build clustering trees and to divide modules. Besides, the correlation between modules and samples was analyzed using WGCNA. The protein-protein interaction (PPI) networks were constructed for *Pyrus* based on the data of *Arabidopsis thaliana* produced by the Search Tool for the Retrieval of Interacting Genes/Proteins database (STRING, https://string-db.org/) and visualized using Cytoscape software (version 3.7.2)^11^. The nodes of the network represented proteins encoded by DEGs and their functional partners in the predicted pairwise interaction network.

### Transcription Factor Identification

Transcription factor (TF) families were identified using the Plant Transcription Factor Database PlantTFDB 4.0^12^ (Jin et al., 2017). The Hmmscan *E*-value and BLAST *E*-value are all set to 1e^–05^. The TF genes were classified into various plant transcription factor families based on conserved domains predicted in the above analysis.

### Quantitative Real-Time PCR Validation

The complementary DNA (cDNA) synthesis was performed on 1 μg of the total RNA (previous RNA-Seq library construction) with the 5× All-In-One RT MasterMix (with AccuRT Genomic DNA Removal Kit; Applied Biological Materials Inc., United States) according to the instructions of the manufacturer. Samples of cDNA were diluted 1:2 to the final template concentration for quantitative Real-time PCR (qRT-PCR) by using CFX96 Real-Time PCR Detection System (Bio-Rad, United States). PCR amplification was performed with 5 μL of iTaq™ Universal SYBR^®^ Green Supermix, 3.5 μL of nuclease-free H_2_O, 1 μL of cDNA, and 10 pmol of forward and reverse primers in a final volume of 10 μL. The PCR cycles are as follows: 95°C for 20 s, followed by 40 cycles of 95°C for 5 s, 56°C for 15 s, and 72°C for 15 s. At the end of the reaction, melt curves were run for all primer pairs to check for dimerization. The *P. pyrifolia beta-adaptin-like gene A* (XM_009369647) was used to normalize the RNA samples for each qRT-PCR and relative expression levels were calculated based on the 2^–ΔΔCT^ method (Schmittgen and Livak, 2008). All qRT-PCR primers are listed in Supplementary Table 1. Each treatment consisted of two biological repeats and three technical replicates.

### Measurement of Phytohormone

The contents of JA and SA were detected using previously described methods at Shanghai Applied Protein Technology Co., Ltd (Shanghai, China) (Shao et al., 2019). Briefly, ∼100 mg pear leaf from each treatment was ground to a fine powder in liquid nitrogen using a mortar and pestle. The plant hormones were extracted from the powder at 4°C for 12 h with 1 mL of ethyl acetate supplemented with internal standards and then centrifuged at 14,000 g for 15 min at 4°C. The supernatant was carefully transferred to a new 1.5 mL tube and the pellet was again extracted with 500 μL of ethyl acetate at 4°C for 1 h. The supernatant from the second extraction was collected and pooled with the first extraction. The supernatant was evaporated to dryness under nitrogen gas flow and then dissolved in 500 μL of 50% (v/v) acetonitrile. After being centrifuged (at 14,000 g and 4°C, for 10 min), the supernatant was then analyzed by high-performance liquid chromatography (HPLC)-electrospray ionization-tandem mass spectrometry. The mobile phase consisted of a combination of solvent A [0.05% (v/v) formic acid in water] and solvent B [0.05% (v/v) formic acid in acetonitrile]. The linear gradient was as follows: 2 to 98% B (v/v) for 10 min, 2% B (v/v) for 10.1 min, and hold at 2% B for 13 min. The mass spectrometer (Qtrap 5500 System, AB Sciex) equipped with an electrospray ionization source was operated in positive/negative ionization and multiple reaction monitoring modes. The MS parameters were as follows: source temperature, 500°C; ion source gas 1, 45 psi; ion source gas 2, 45 psi; curtain gas, 30 psi; and ion spray voltage, 5500 V.

### Statistical Analysis

Statistical analysis was conducted with IBM SPSS Statistics 21.0 software (IBM Corp. Released 2012. IBM SPSS Statistics for Windows, Version 21.0. Armonk, NY: IBM Corp.) by one-way ANOVA, and means were compared using Duncan’s test at a significance level of *P* = 0.05. The homogeneity of variance was tested before analysis.

## Results

### Symptoms Induced by Two Pathogenetic Strains of *Colletotrichum fructicola*

**FIGURE 1:**
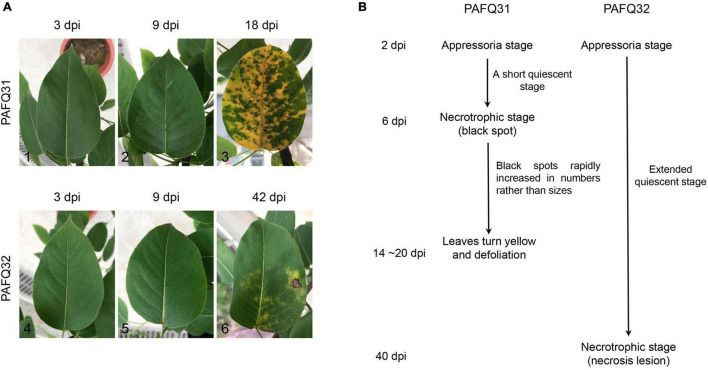
The representative serial symptoms on *Pyrus pyrifolia* leaves induced by two *Colletotrichum fructicola* strains under unwounded conditions and the deduced pathogenetic stages. **(A)** The representative symptoms at 3, 9, and 18 days post-inoculation (dpi) for strain PAFQ31 (A1–A3), and at 3, 9, and 42 dpi for strain PAFQ32 (A4–A6), respectively. **(B)** The deduced pathogenetic stages for strains PAFQ31 and PAFQ32.

Two pathogenetic strains of *C. fructicola* identified in our previous study (Fu et al., 2019), PAFQ31 and PAFQ32 causing TS and BnL on pear leaves, respectively, were selected for inoculation on attached pear leaves (*P. pyriforia* cv. Cuiguan) under the unwounded conditions. As expected, strain PAFQ31 caused TS symptoms at 6 days post-inoculation (dpi), with numbers of the tiny spots rapidly increasing and spreading to the entire leaf area in the following days (Figure 1A2; 9 dpi). At 14–20 dpi, the green parts neighboring the TS started yellowing, subsequently, the veins and petioles turned yellow, and finally, severe defoliation was observed (Figure 1A3). In contrast, strain PAFQ32 did not induce small brown necrotic lesions until 40 dpi, and subsequently, the small lesions expanded into BnL symptoms under appropriate conditions but no defoliation was observed for these diseased leaves (Figure 1A6). In parallel, no lesions were observed on the leaves as inoculated with sterile water. The symptomatic samples were subjected to fungal isolation at the parts neighboring the asymptomatic regions, making colonies matching the inoculated ones according to their morphologies and ITS sequences.

To get insight into the mechanism under the symptoms induced by each strain, the inoculated pear leaves were collected in triplicates for transcriptome sequencing at latent (QS) and evident (NS) stages (Figure 1B), i.e., at 4 and 9 dpi for strain PAFQ31, and 4 and 42 dpi for strain PAFQ32, respectively. Pear leaves without inoculation at QS were involved in parallel as the controls, respectively. In total, 15 RNA samples were prepared for cDNA library construction and sequencing.

### Transcriptome Profiling and Differential Expression Genes Identification

In total, 7.0–11.3 Gb of high-quality clean data were obtained from each leaf sample (Supplementary Table 2). The clean reads were aligned to the pear reference genome (*P. bretschneideri* cv. Suli genome; accession no. PRJNA259338) (Wu et al., 2013), with over 76% reads mapped to the reference genome (Supplementary Table 2). To evaluate the correlations of three independent biological replicates in each treatment and eliminate the possible outliers, principal component analysis (PCA) and hierarchical cluster analysis were carried out for the obtained data and an outlier sample from each treatment was abandoned (Supplementary Figure 1A). Finally, ten samples with high correlations between the biological replicates (*R*^2^ = 0.969–0.982) were subjected to further analyses (Supplementary Figure 1B).

**FIGURE 2:**
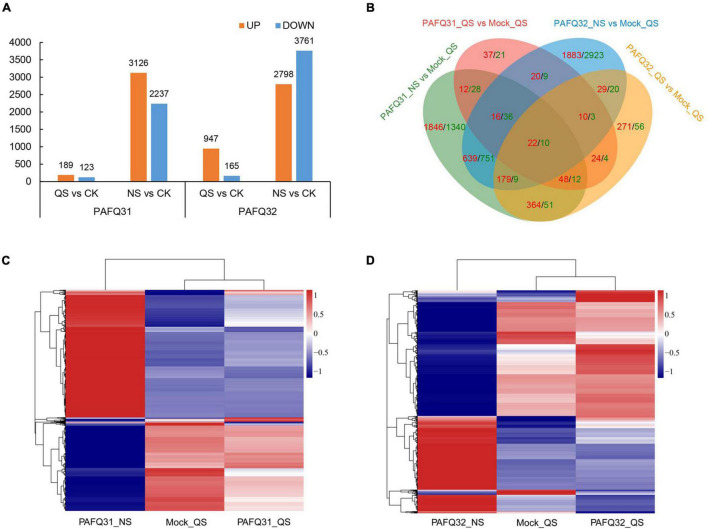
Transcriptome analysis of pear leaves at quiescent stage (QS) and necrotrophic stage (NS) after inoculation by two *C. fructicola* strains. **(A)** Statistical analysis of upregulated and downregulated differential expression genes (DEGs) in response to the infection of both *C. fructicola* strains at QS and NS, respectively. **(B)** Venn diagrams of the upregulated and downregulated DEGs of *P. pyrifolia*, indicated by red and green color, respectively, as compared with controls for each strain. **(C,D)** Heatmap analysis for 5,485 and 7,122 DEGs with at least a twofold expression differences with a *p*-value < 0.05 as in response to the infection of strain PAFQ31 **(C)** and PAFQ32 **(D)**, respectively. The bars indicate the standardized log_2_(FPKM) accompanies the expression profile, ranging from navy (-1) to white (0) to firebrick3 (1).

Based on the DESeq2 analysis, a total of 5,485 (312 in QS and 5,363 in NS) and 7,122 (1,112 in QS and 6,559 in NS) DEGs (no less than twofold change) were revealed in the leaves of *P. pyrifolia*, with 3,217 (189 in QS and 3,126 in NS) and 3,505 (947 in QS and 2,798 in NS) upregulated in the leaves infected by strain PAFQ31 and PAFQ32 as compared with the controls, respectively (Figure 2A). Venn diagram showed that most of the DEGs were uniquely expressed, and far less overlapped in response to the infection of each strain, with the numbers significantly higher at NS than those at QS (Figure 2B). Of which, only 22 up- and 10 downregulated DEGs were observed in both PAFQ31- and PAFQ32-infected leaves during the infection progress, whereas 3,284 (98 in QS and 3,226 in NS) and 5,182 DEGs (376 in QS and 4,855 in NS) were uniquely expressed in the PAFQ31- and PAFQ32-infected leaves, respectively (Figure 2B). The heatmap analysis further reveals that the DEGs were in a very small amount at QS while dramatically increasing their numbers at NS triggered by each strain, indicating a clear process of *P. pyrifolia* in response to the fungal infection, from less apparent to dramatically strong along with the infection of *C. fructicola* (Figures 2C,D).

### Enrichment Analyses of Pear Leaves in Response to Two *Colletotrichum fructicola* Strains

**FIGURE 3:**
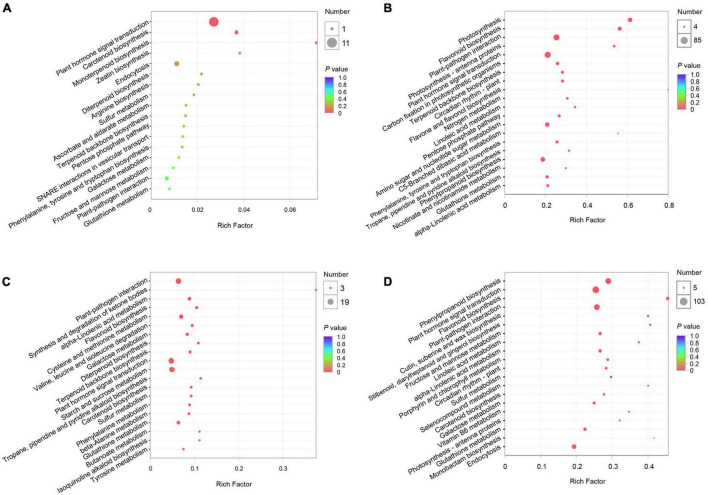
Kyoto Encyclopedia of Genes and Genomes (KEGG) pathway enrichment analyses of DEGs of *P. pyrifolia* at QS and NS in response to the infection of two *C. fructicola* strains. **(A,B)** KEGG pathway enrichment of DEGs response to strain PAFQ31 at QS **(A)** and NS **(B)**, respectively. **(C,D)** KEGG pathway enrichment of DEGs response to strain PAFQ32 at QS **(C)** and NS **(D)**, respectively.

To characterize the pear response to two *C. fructicola* strains, we performed KEGG and GO enrichment analyses of the DEGs induced by each strain at QS and NS, respectively. The KEGG enrichment analyses revealed 312 and 5,363 DEGs enriched in 26 and 115 pathways at QS and NS in *P. pyrifola* leaves as infected by strain PAFQ31, respectively. Of these, only small numbers of DEGs were enriched in plant hormone signal transduction (11 genes; ko04075) and carotenoid biosynthesis (2; ko00906) at QS (Figure 3A), whereas large numbers of genes in plant hormone signal transduction (85; ko04075), plant-pathogen interaction (76; ko04626), phenylpropanoid biosynthesis (50; ko00940), and flavonoid biosynthesis (32; ko00941) metabolic pathways at NS (Figure 3B). Notably, photosynthesis (46; ko00195), carbon fixation in photosynthetic organisms (25; ko00710), and photosynthesis-antenna proteins (15; ko00196) were also significantly enriched at NS (Figure 3B).

As infected by strain PAFQ32, 1,112 and 6,559 DEGs were enriched in 82 and 115 pathways at QS and NS, respectively (Figures 3C,D), with most of the defense-related pathways the same as those of PAFQ31. Besides, stilbenoid, diarylheptanoid, and gingerol biosynthesis (13; ko00945), cutin, suberine, and wax biosynthesis (16; ko00073), porphyrin and chlorophyll metabolism (19; ko00860), and carotenoid biosynthesis (15; ko00906) were also highly enriched at NS (Figure 3D). It is worthy to note that, multiple defense-related pathways were only activated at QS by the infection of PAFQ32 instead of PAFQ31 (Supplementary Table 3). Instead, more of these DEGs at NS are related to the infection by PAFQ31 rather than by PAFQ32. For GO enrichment, the DEGs at NS were mainly enriched in similar metabolic processes for each strain but uniquely enriched in photosynthesis (GO:0015979), photosystem (GO:0009521), photosystem II (GO:0009523), and photosystem II oxygen-evolving complex (GO:0009654) for PAFQ31 infection, suggesting the infection of strain PAFQ31 seriously affect the photosynthesis of plants (Supplementary Figure 2).

### Different Plant Hormone Pathways Activated in Response to Two *Colletotrichum fructicola* Strains

**FIGURE 4:**
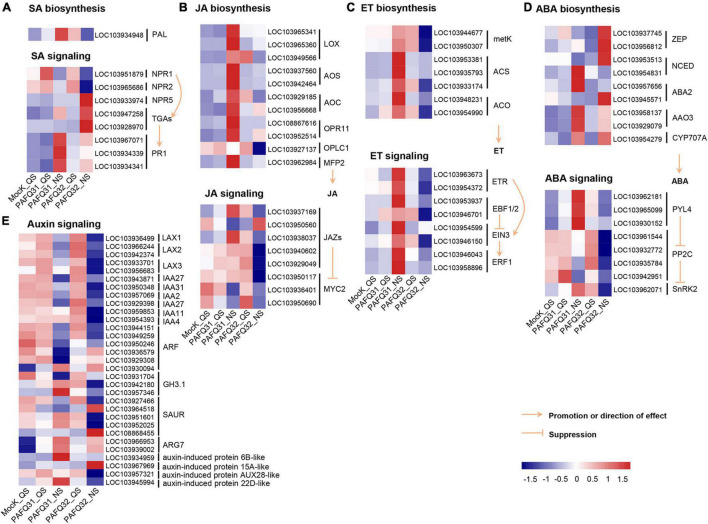
Heatmap analyses of DEGs related to phytohormone signal transduction pathways in the leaves of *P. pyrifolia* in response to *C. fructicola* infection. **(A–E)** Heatmap of the DEGs involved in the salicylic acid (SA), jasmonic acid (JA), ethylene (ET), abscisic acid (ABA), and auxin pathway, respectively.

The related genes were further analyzed because large DEG numbers were significantly enriched into plant signal transduction pathways. Of which, homologous *non-expresser of pathogenesis-related genes* (*Pp-NPR1* and *Pp-NPR2*), the key regulators of plant SA-mediated defense response, were mainly upregulated triggered by each strain at QS, whereas the genes at the downstream of SA signaling pathway such as *Pp-TGAs* (*Pp-TGA2.3*, *Pp-TGA4*) and *pathogenesis-related gene 1* (*Pp-PR1*) were upregulated at NS, with higher accumulation levels in the PAFQ31-infected leaves than those in the PAFQ32-infected (Figure 4A). Additionally, the expression of genes involved in the JA, ET, and ABA signaling pathways was specifically upregulated in the PAFQ31-infected leaves (Figures 4B–D). Moreover, the expression levels of genes related to the hormone biosynthesis pathway (the upstream pathway of the hormone signal transduction) were consistent with these related to hormone signal transduction pathways (Figures 4A–D).

The genes related to the auxin signaling pathway, e.g., LAX1 (LIKE-AUXIN1), LAX2, LAX3, IAA2 (auxin-responsive gene), IAA27, and IAA31, were constant at QS while downregulated at NS after the infection by each strain (Figure 4E). In contrast, several genes (LOC103966953, LOC103939002, LOC103934959, LOC103967969, LOC103945994) encoding indole-3-acetic acid (IAA)-induced protein ARG7-like or auxin-induced protein were upregulated at NS after infection by each strain (Figure 4E). Moreover, genes (*Gretchen Hagen3.1*, *GH3.1*; LOC103942180, LOC103957346) encoding IAA-amido synthetase GH3.1 were obviously upregulated in the PAFQ31-infected leaves at NS, while downregulated in the PAFQ32-infected (Figure 4E).

### Phenylpropanoid Biosynthesis Activated at Necrotrophic Stage of *Colletotrichum fructicola* Infection

**FIGURE 5:**
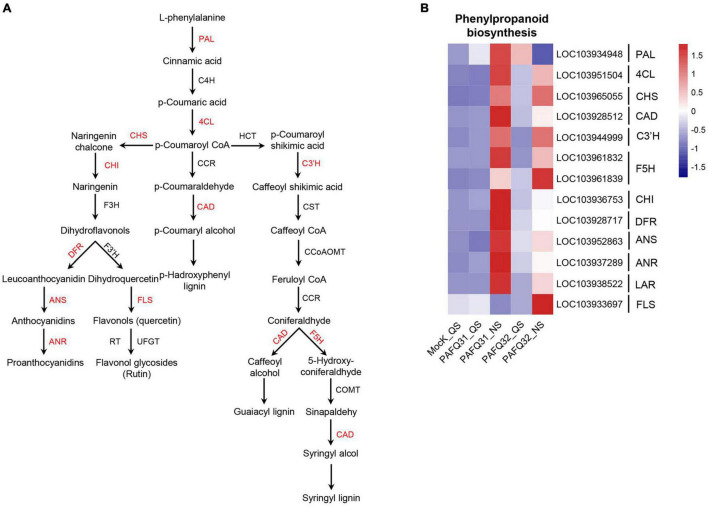
Heatmap analyses of the DEGs related to phenylpropanoid metabolism pathway in *P. pyrifolia* leaves in response to the infection of each *C. fructicola* strain. **(A)** Simplified diagram of phenylpropanoid metabolism pathway, with red color indicating the upregulated genes. **(B)** Expression profiles of the DEGs related to phenylpropanoid metabolism pathway.

To check whether the inoculated leaves behave defensively against the fungal damage, the genes involved in phenylpropanoid metabolism (Figure 5A), an important pathway of secondary metabolism in plants closely related to disease resistance (Wang et al., 2018; Sudheeran et al., 2021), were subjected to an analysis of their expression levels. The related genes, including homologous *phenylalanine ammonia lyase* (*Pp-PAL*, LOC103934948) gene, were significantly upregulated at QS or NS after infection by each strain (Figure 5B). Additionally, the genes involved in lignin biosynthesis, including *4-coumarate: coenzyme A ligase* (*Pp-4CL*, LOC103951504), *cinnamyl alcohol dehydrogenase* (*Pp-CAD*), *p-coumarate 3-hydroxylase* (*Pp-C3’H*), and *ferulate 5-hydroxylase* (*Pp-F5H*) gene, were constant at QS but upregulated at NS as compared with the controls (Figure 5B). However, the genes related to anthocyanin synthesis (such as *chalcone isomerase*/*Pp-CHI*, LOC103936753; *dihydroflavanol 4-reductase*/*Pp-DFR*, LOC103928717; *anthocyanidin synthase*/*Pp-ANS*, LOC103952863; *anthocyanidin reductase*/*Pp-ANR*, LOC103937289) were significantly upregulated in the PAFQ31-infected leaves (Figure 5B).

### Co-expression Modules in Pear Leaves Associated With Two *Colletotrichum fructicola* Strains

**FIGURE 6:**
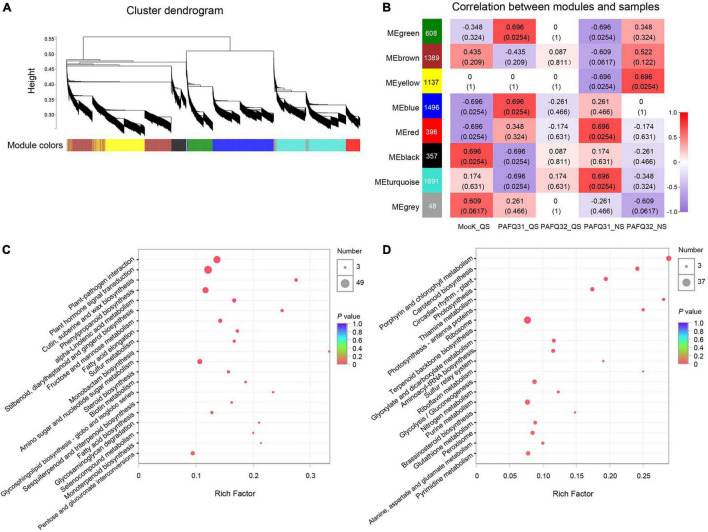
WGCNA for the DEGs of *P. pyrifolia* at QS and NS in response to *C. fructicola* infection. **(A)** Gene co-expression modules were identified for the DEGs for the infection of both *C. fructicola* strains. Different colors represent different gene modules. **(B)** Association between modules and samples. The numbers in each cell are the correlation coefficient (top) and *P*-value (bottom). A colored bar indicating the level of correlations between modules and samples. **(C)** KEGG pathways based on the ‘MEyellow’ and ‘MEbrown’ modules. **(D)** KEGG pathways based on the ‘MEred’ and ‘MEturquoise’ modules.

To determine the gene regulatory network in pear leaves in response to the *C. fructicola* infection, a WGCNA was constructed using 7,122 DEGs (after pre-process) in the leaves infected by strains PAFQ31 and PAFQ32. In total, eight gene co-expression modules, containing genes from 48 to 1,691, were identified according to correlations between gene expression levels (Figure 6A). It reveals that each module was correlated with a particular stage (Figure 6B), and some were specifically related to the strains. Of which, the modules ‘MEred’ and ‘MEturquoise’ were highly associated with the infection of strain PAFQ31 at NS, whereas the ‘MEyellow’ and ‘MEbrown’ with strain PAFQ32 at the same stage. KEGG enrichment analyses reveal that the genes in modules ‘MEyellow’ and ‘MEbrown’ were highly enriched in plant-pathogen interaction, plant hormone signal transduction, and phenylpropanoid biosynthesis metabolic pathways (Figure 6C), with the expression patterns as previously indicated (Figures 4, 5 and Supplementary Figures 3A,B). For the modules ‘MEred’ and ‘MEturquoise’, their genes were related to porphyrin and chlorophyll metabolism, carotenoid biosynthesis, circadian rhythm–plant (ko04712), photosynthesis–antenna proteins, photosynthesis, thiamine metabolism (ko00730), sulfur relay system (ko04122), and riboflavin metabolism (ko00740) (Figure 6D). The genes involved in chlorophyll metabolism, carotenoid biosynthesis, and photosynthesis pathways showed different expression patterns between both strains, which were significantly downregulated in the PAFQ31-infected leaves, while upregulated in the PAFQ32-infected (Supplementary Figures 3C–E).

**FIGURE 7:**
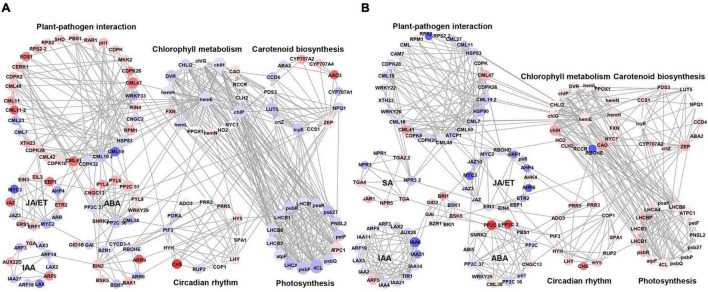
Protein-protein interaction network for the DEGs of defense-related pathways in *P. pyrifolia* at NS after infection by each *C. fructicola* strain. **(A,B)** The network constructed for strain PAFQ31 and PAFQ32, respectively. Red and purple indicate up-and downregulated gene expression, respectively.

To better understand the phenomenon of early defoliation caused by strain PAFQ31, 225 DEGs of PAFQ31_NS and 243 DEGs of PAFQ32_NS related to plant-pathogen interaction, plant hormone signal transduction, chlorophyll metabolism, carotenoid biosynthesis, photosynthesis, and circadian rhythm pathways were subjected to build PPI networks as referred to the ones in *Arabidopsis*, respectively (Figure 7). It reveals that most proteins involved in the ET and ABA signaling pathways were upregulated in the PAFQ31-infected leaves, and interacted with the PTI/ETI-related proteins; while the genes involved in the JA and ET signaling pathway were suppressed in the PAFQ32-infected, and the PTI/ETI-related proteins were interactive with the proteins of the SA signaling pathway.

Moreover, we found that several proteins involved in phytohormone signaling, PTI, and ETI are associated with chlorophyll metabolism, carotenoid biosynthesis, or circadian rhythm-related proteins (Figure 7A). Protein phosphatase 2C (PP2C) in ABA signaling interacts with zeaxanthin epoxidase (ZEP) in carotenoid biosynthesis and magnesium-chelatase subunit chlH in chlorophyll metabolism. Moreover, uroporphyrinogen decarboxylase 1 (hemE) in chlorophyll metabolism interacts with different kinds of calmodulin-like proteins (CMLs) and proteins in photosynthesis, likely building a bridge between the plant-pathogen interaction pathway and photosynthesis. By analyzing the expression levels of the corresponding genes of these proteins, it revealed that some of the CMLs interacting with hemE were upregulated and downregulated in the pear leaves after infection by PAFQ31 and PAFQ32, respectively, while corresponding genes related to chlorophyll metabolism pathway and photosynthesis behaved, on the contrary, indicating that these CMLs may serve as key components involved in regulating chlorophyll metabolism and photosynthesis in pear plants and related to the anthracnose-associated early defoliation of pear plants.

### Specific Transcription Factors Involved in the PAFQ31-Associated Early Defoliation

To check whether some TFs were involved in the response to the fungal infection, all DEGs were subjected to TF prediction and TF family analysis. A total of 361 and 440 TFs, belonging to 31 and 37 TF families were identified at NS in the leaves infected by strain PAFQ31 and PAFQ32, respectively, and most of the TFs belong to five families, including AP2/ERF, WRKY, bHLH, MYB, and NAC (Supplementary Figure 4A). In total, 34 and 35 TFs belonging to the WRKY family, one of the largest transcription regulator families in plants related to PTI through MAPK signaling cascade regulation and ETI through R protein (Li et al., 2016), were differentially expressed at NS in the leaves infected by strains PAFQ31 and PAFQ32, respectively, with obviously higher expression levels in the PAFQ31-infected leaves (Supplementary Figure 4B). Of these, 21 genes encoding nine WRKY factors (WRKY9, 44, 45, 51, 53, 55, 65, 70, and 75) were significantly upregulated at NS induced by PAFQ31 infection (Supplementary Table 4). It is worth noting that WRKY45, WRKY53, and WRKY75 have been characterized as positive regulators of leaf senescence (Miao et al., 2004; Chen et al., 2017; Guo et al., 2017). Additionally, other TFs, e.g., TF family NAC also known as an immune or leaf senescence regulator, were also differentially expressed at NS in the PAFQ31-infected leaves (Supplementary Figure 4C).

### Validation of RNA-Seq Data by Quantitative Real-Time PCR and Phytohormone Production Measurements

**FIGURE 8:**
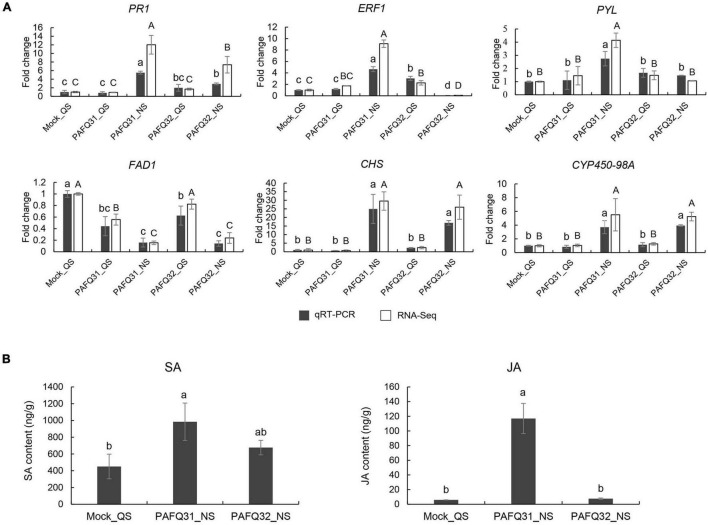
Validation of RNA-Sequencing (RNA-Seq) data by quantitative real-time -PCR (qRT-PCR) and phytohormone production measurements. **(A)** qRT-PCR was conducted with *beta-adaptin-like gene A* (*BETAA-AD*) as an internal reference gene based on six DEGs, including pathogenesis-related protein 1-like (*Pp-PR1*), ethylene-responsive transcription factor 1B-like (*Pp-ERF1*), abscisic acid receptor PYL4-like (*Pp-PYL*), delta(8)-fatty-acid desaturase 1-like (*Pp-FAD1*), chalcone synthase (*Pp-CHS*), and cytochrome P450 98A2-like (*Pp-CYP450-98A*). Error bars represent the standard deviations of the mean. Different letters over the error bars indicate the significant difference at the *P* = 0.05 level. The results of significance analysis for RNA-Seq data and qRT-PCR data are showed by **(A–C)** and **(a–c)**, respectively. **(B)** The levels of SA and JA in *P. pyrifolia* infected by strains PAFQ31 and PAFQ32 at NS, respectively. Error bars represent the standard deviations of the mean. Different letters over the error bars indicate the significant difference at the *P* = 0.05 level.

To verify the transcriptome sequencing data, six DEGs were randomly chosen for qRT-PCR analysis. The results showed that all the selected genes exhibited expression patterns similar to those obtained by RNA-Seq, supporting the transcriptome sequencing results (Figure 8A). Correspondingly, gene *Pp-PR1* showed significantly higher expression levels at NS in the leaves infected by both strains compared to the healthy leaves, while genes *Pp-ERF1* and *Pp-PYL* were specifically upregulated at NS in the PAFQ31-infected leaves.

To further confirm the phytohormone accumulation triggered by the infection of each strain, endogenous SA and JA were measured in the pear leaves at NS. The results showed that SA accumulation slightly increased (without significance) after infection by both PAFQ31 and PAFQ32 (Figure 8B). JA accumulation was obviously higher in the PAFQ31-infected leaves than in the PAFQ32-infected (Figure 8B).

## Discussion

*Colletotrichum fructicola* commonly induces sunken BnL on leaves or BrL on fruits on many important woody plants, e.g., *Camellia* spp., *Citrus* spp., *Malus* spp., *Mangifera* spp., and *Pyrus* spp. (Huang et al., 2013; Lima et al., 2013; Jiang et al., 2014; Liu et al., 2015; Munir et al., 2016; Wang et al., 2018). In *Camellia oleifera*, it could shift from intracellular hemibiotrophy to a destructive necrotrophic stage in the diseased process as observed through histopathological and ultrastructural approaches (Li et al., 2021). Similarly, both intracellular and subcuticular intramural infections were also observed in apple leaves during the biotrophic development stage (before 36 h post inoculation) as infected by *C. fructicola* (Shang et al., 2020), which caused TS symptoms accompanied by severe defoliation of apple plants similar to those of the pear plants observed in this study (Zhang et al., 2015; Fu et al., 2019; Shang et al., 2020). Unlike hemibiotrophic *Colletotrichum* strains, the necrotrophic ones only have a very short biotrophic phase in the host (Perfect et al., 1999; Amil-Ruiz et al., 2016; De Silva et al., 2017). In this study, inoculation experiments showed that *C. fructicola* strain PAFQ31 induced TS at 6 dpi after a short time of QS on the leaves of *P. pyrifolia*, whereas strain PAFQ32 caused BnL symptoms as long as 40 days (Figure 1A). Moreover, our previous inoculations showed that strain PAFQ32 was difficult to induce apparent symptoms on *P. pyrifolia* leaves under unwounded conditions (Fu et al., 2019). These results indicated that PAFQ31 is most likely a necrotrophic strain, while PAFQ32 is a hemibiotrophic one.

To understand the molecular mechanism underlying the host response and the diverse symptoms, two *C. fructicola* pathogenetic strains (PAFQ31 and PAFQ32) were inoculated on *P. pyrifolia* leaves and subjected to transcriptome sequencing at QS and NS. RNA-Seq results revealed that the gene profiles were significantly changed along with the infection time, with an obviously higher number of DEGs at NS than that at QS, corresponding to the symptom change from the latent to the evident. In general, host defense reactions would initiate or intensify during the early stage or the QS of *Colletotrichum* infection (Alkan et al., 2015; Zhang et al., 2018). Here, we found that PAFQ32 induced the up-regulation of the gene expression of defense-related metabolic pathways in the host at QS, while the pear leaves did not initiate strong defense responses against PAFQ31 infection at this stage (Supplementary Table 3), probably because PAFQ31 only colonized under the cuticle within the cell wall around the epidermis without penetrating the protoplasts. At NS, the transcriptomic analysis reveals a wide range of metabolism pathway changes in response to the infection of both *C. fructicola* pathogenetic strains, including SA, JA, ET, and ABA biosynthesis and signaling, phenylpropanoid metabolism, etc., with clearly significant differences in most of these pathways induced by both infections.

Plant hormones play key roles in triggering the plant immune signaling network (Pieterse et al., 2012). SA signaling is well known to be the center player for the plant against biotrophic/hemibiotrophic pathogens, while JA signaling is essential for the response to necrotrophic pathogens (Bari and Jones, 2009). The SA signaling pathway is also involved in the host defense against necrotrophic fungi since they always have a very short biotrophic phase before killing the host tissues with their secondary hyphae (De Silva et al., 2017; Wang Q. et al., 2019; Liu et al., 2020; Zhao et al., 2020). *NPR1*, ahead of *PR*, is a key regulator of plant SA-mediated defense response. Example, overexpression of *NPR1* derived from *A. thaliana* in carrot, tomato, and strawberry plants stimulates a broad-spectrum resistance to fungi, bacteria, or viruses (Lin et al., 2004; Wally et al., 2009; Silva et al., 2015); overexpression of *CmNPR1* in *Chrysanthemum morifolium* increased plant resistance to a necrotrophic fungus, *Alternaria* spp. (Zhao et al., 2020). In recent years, several studies have shown that the SA signaling pathway is likely to be the core defense mechanism of plants against *Colletotrichum* species (Alkan et al., 2015; Zhang et al., 2018; Shi et al., 2019); the JA signaling pathway together with the partial SA signaling pathway were activated in strawberries against the infection of *C. acutatum*, a subcuticular intramural invasion in strawberries (Arroyo et al., 2005; Amil-Ruiz et al., 2016). Consistent with the previous studies, *Pp-NPR1* and other genes related to SA biosynthesis and signaling were significantly upregulated in pear leaves along with the infection by both *C. fructicola* strains (Figure 4A). However, in our study, the genes involved in the JA, ET, and ABA biosynthesis and signaling pathways were also specifically upregulated in the PAFQ31-infected leaves (Figures 4B–D). These results suggest that *P. pyrifolia* defense systems are generally activated by the infection of *C. fructicola* strains, but with different mechanisms in response to the different pathogenetic strains, further suggesting that the two strains may have different lifestyles.

Auxin is a group of molecules (including IAA) that not only acts as a growth hormone mediating apical growth and development but also plays a central role in balancing plant resistance responses (Denancé et al., 2013), which could negatively impact plant defense by suppressing SA levels and signaling (Robert-Seilaniantz et al., 2011; Pieterse et al., 2012; Svoboda et al., 2021). Some fungal pathogens, e.g., *Fusarium oxysporum*, *F. arthosporioides*, and *C. gloeosporioides* f. sp. *aeschynomene*, are able to secrete auxin into host plants to enhance their susceptibility (Cohen et al., 2002; Maor et al., 2004). In counterpart, some plants encode GH3 proteins to suppress auxin production, thus, enhancing the resistance against the pathogenetic fungi, as exemplified by GH3.1 and GH3.2 in *Oryza sativa* and GH3.2 and GH3.4 in *A. thaliana*, *Nicotiana benthamiana*, and tomato (Domingo et al., 2009; Fu et al., 2011). In this study, the auxin-related genes such as LIKE-AUXIN genes (LAXs) and IAAs were significantly downregulated at NS after infection by each strain (Figure 4E); genes *GH3.1* were particularly upregulated after PAFQ31 infection (Figure 4E). The results may partly explain why SA levels were highly accumulated in the infected leaves.

Phenylpropanoid metabolism, which can produce phytoalexin, lignin, flavonoid, and anthocyanin, is closely related to plant defense against pathogens (Wang et al., 2018). In this study, some DEGs were enriched in the biosynthesis of flavonoid and phenylpropanoid. Further analysis revealed that the genes related to lignin and flavonoid biosynthesis were significantly upregulated at NS in the leaves infected by each of the two strains, indicating that phytoalexin and lignin comprise an important defense pathway employed by *P. pyrifolia* in response to *C. fructicola* infection. Moreover, phenylpropanoid derivatives also serve as potential substrates for PODs activity (Alkan et al., 2015). Here, the significant up-regulation of *Pp-POD* genes at NS (Supplementary Figure 3A) may also indicate they cooperate to activate the defense mechanism of pear plants. In general, plants can synthesize SA through the phenylalanine ammonia lyase (PAL) and isochorismate (IC) pathways, in which the IC pathway plays a major role in pathogen-induced SA synthesis (Ding and Ding, 2020). In this study, the upregulated expression of *PAL* may also contribute to SA biosynthesis for plant defense. However, the degree of *PAL* upregulated expression in plants after inoculation of strains PAFQ31 and PAFQ32 were different, which may be a reason for the difference in SA accumulation level (Figure 8B) in the leaves infected by each of the two strains.

As a very intricate and exquisite physiological process, senescence is a necessary stage in the natural development of plants and is controlled by a series of internal factors (Guo and Gan, 2012). It is also affected by a variety of environmental factors including pathogen infection (Guo and Gan, 2005). The most obvious sign of leaf senescence is the change of leaf color due to macromolecule breakdown, chlorophyll degradation, and the change of endogenous hormone content (Kusaba et al., 2013; Woo et al., 2019). Plant hormones also act a key role in controlling the progression of leaf senescence. ABA, ET, JA, and SA are considered as senescence promotors, while auxins, CKs, and GAs are senescence suppressors (Guo and Gan, 2012; Estornell et al., 2013). For example, the SA and ABA-related genes in *A. thaliana* were significantly upregulated in normal leaves as compared with the senescing ones (van der Graaff et al., 2006; Breeze et al., 2011); the transcriptome analysis of the calyx abscission zone of Huanglongbing-diseased sweet orange revealed that ET and JA signaling are involved in regulating the fruit abscission (Zhao et al., 2019). In this study, besides the fact that multiple defense-related metabolic pathways were stimulated against the fungal infection in the pear leaves, the photosynthesis-related DEGs were uniquely enriched in the PAFQ31-infected leaves (Figures 3B, 6D and Supplementary Figure 2A). Moreover, two modules (MEred and MEturquoise) closely related to the PAFQ31-infected leaves were significantly enriched into the photosynthesis, chlorophyll metabolism, carotenoid biosynthesis, and circadian rhythm, and their related involved in these pathways were significantly downregulated in these leaves at NS (Supplementary Figures 3C–E). These changes correspond to the leaves turning yellowish at this stage, and it supports a conclusion that PAFQ31 infection caused chlorophyll to be degraded and photosynthesis inhibited, resulting in a rapid leaf yellowing (14–20 dpi; Figure 1A3), since chlorophylls are essential molecules responsible for photosynthesis in photosynthetic organisms (Tanaka and Tanaka, 2006), and the degradation of chlorophylls concomitant with chloroplast disassembly and the loss of ability to photosynthesize directly changed leaf color from green to yellow (Woo et al., 2019). Furthermore, the pathways related to leaf senescence and defoliation were also revealed in PAFQ31-infected leaves: (1) The JA, ET, and ABA biosynthesis and signaling pathways were activated, which may also induce the upregulated expression of senescence-associated genes (SAGs); (2) The PPI analysis demonstrated that important proteins in the ABA, JA, and ET signaling pathways, such as protein PP2C, MYC2 and ERF1, interact directly or indirectly with proteins related to the chlorophyll metabolism and photosynthesis pathways; (3) the homologous CNGC2, which may be involved in the regulation of leaf senescence by regulating chlorophyll metabolism since Ca^2+^ is a key component involved in plant senescence signaling such as *A. thaliana* cyclic nucleotide-gated channel (AtCNGC2) (Ma et al., 2010), interacted with red chlorophyll catabolite reductase (RCCR) in the chlorophyll metabolism in the PPI network (Figure 7); (4) multiple types of CMLs interact with hemE related to chlorophyll metabolism pathways, and their expression levels showed opposite trends between both strain’s infection; (5) some senescence-related TFs, WRKY45, WRKY53, WRKY75, NAC29, and NAC72, showed upregulated expression only in the leaves infected by PAFQ31 instead of PAFQ32 (Supplementary Table 4), since the TFs had been shown to be involved in leaf senescence (Miao and Zentgraf, 2007; Woo et al., 2019). Correspondingly, the leaves fell off at the later stage after infection by PAFQ31 instead of PAFQ32. These data provide a clear clue that JA, ET, and ABA accumulated in the PAFQ31-infected leaves, which negatively affected the chlorophyll metabolism and photosynthesis pathways but positively affected the expression of senescence-associated TFs and genes, resulting in leaf yellowing and defoliation; whereas SA inhibited JA-induced gene expression in the PAFQ32-infected leaves, which led to hypersensitive response-like reaction and BnL symptoms.

**FIGURE 9:**
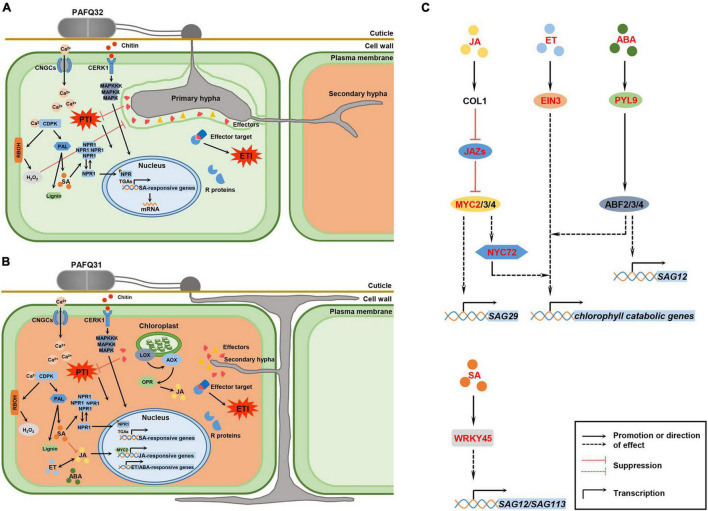
Hypothetical model for *P. pyrifolia* in response to the infection of two pathogenetic *C. fructicola* strains and the crosstalk between hormone signaling and transcription factors (TFs) that are involved in leaf senescence. **(A,B)** Proposed models for *P. pyrifolia* in response to the infection of strain PAFQ32 and PAFQ31, respectively. In panel **(A)**, (1) After infection by strain PAFQ32, pear plants sense fungal pathogen-associated molecular patterns (PAMPs) (exemplified by chitin) with host pattern recognition receptors, and produce a variety of defense responses including activation of the mitogen-activated protein kinases (MAPKs) and plant hormones signal transduction pathway, change of intracellular Ca^2+^ concentration, and production of reactive oxygen species (ROS); (2) The fungus further secrets multiple effectors to target host proteins and suppress pathogen-associated molecular patterns (PAMPs)-triggered immunity (PTI); (3) The plants synthesize resistance (R) proteins to recognize effector activity that triggered strong effector-triggered immune responses; (4) SA may act a pivotal role in the process of PTI or effector-triggered immunity (ETI) activation. In panel **(B)**, (1) strain PAFQ31 grows under the cuticle within the cell wall around the epidermis and shortly form thin secondary hyphae to destruct the colonized host tissues; (2) The plants active PTI or ETI, and JA, ET, and ABA signaling pathways. **(C)** Effects of specific phytohormones and TFs on leaf senescence. After PAFQ31 infection, pear plants activated JA, ET, ABA, and SA signaling pathways, which may induce the upregulated expression of senescence-associated genes (SAGs) and chlorophyll catabolic genes, and finally result in defoliation of the diseased leaves. Phytohormone, protein and TFs marked in red indicated that the expression of related genes were upregulated in this study. Solid lines represent well-defined biological processes, and dotted lines represent the information reported in the studies of *Arabidopsis thaliana* as a reference (Woo et al., 2019).

This study implemented comparative transcriptome analyses of the *P. pyrifolia* leaves infected by *C. fructicola*, revealing that multiple biological processes were stimulated in *P. pyrifolia* in response to the infection of two *C. fructicola* pathogenetic strains (Figures 9A,B), such as the activation of PTI and ETI, plant hormones, and metabolism of flavonoids and phenylpropane. Importantly, this study reveals specific genes, pathways, phytohormones, and TFs associated with the diverse symptoms of pear leaves caused by *C. fructicola* infection (Figure 9C), which may contribute to understanding the molecular mechanism of two diverse leaf symptoms of pear plants caused by two *C. fructicola* pathogenetic strains, and then the pathogenetic process of *Colletotrichum* fungi in plants.

## Data Availability Statement

The datasets presented in this study can be found in online repositories. The names of the repository/repositories and accession number(s) can be found below: https://www.ncbi.nlm.nih.gov/, PRJNA698408.

## Author Contributions

GW and NH conceived the study. MF performed the experiment. MF and QB analyzed the data and wrote the manuscript. HZ, YG, YP, PZ, and LS prepared the experimental materials. WX and GW revised the manuscript. All authors read and approved the final manuscript.

## Conflict of Interest

The authors declare that the research was conducted in the absence of any commercial or financial relationships that could be construed as a potential conflict of interest.

## Publisher’s Note

All claims expressed in this article are solely those of the authors and do not necessarily represent those of their affiliated organizations, or those of the publisher, the editors and the reviewers. Any product that may be evaluated in this article, or claim that may be made by its manufacturer, is not guaranteed or endorsed by the publisher.

## Footnotes

1
https://github.com/jstjohn/SeqPrep

2
https://github.com/najoshi/sickle

3
https://www.ncbi.nlm.nih.gov/genome/?term=Pyrus%20bretschneideri

4
https://www.rdocumentation.org/packages/Rsubread/versions/1.22.2/topics/featureCounts

5
https://bioconductor.org/packages/release/bioc/html/DESeq2.html

6
http://www.omicshare.com/tools

7
https://github.com/tanghaibao/Goatools

8
http://kobas.cbi.pku.edu.cn/kobas3/?t=1

9
www.majorbio.com

10
https://horvath.genetics.ucla.edu/html/CoexpressionNetwork/Rpackages/WGCNA/

11
https://cytoscape.org/

12
http://planttfdb.gao-lab.org/
