# Supplementary material for: Transcriptome Analysis of the Molecular Patterns of Pear Plants Infected by Two Colletotrichum fructicola Pathogenic Strains Causing Contrasting Sets of Leaf Symptoms
Source: Front Plant Sci. 2022 Feb 16;13:761133. doi: 10.3389/fpls.2022.761133 (PMC8888856; doi:10.3389/fpls.2022.761133)
Supplement: Supplementary file 1 [file Presentation_1.PPTX]

## Slide 1
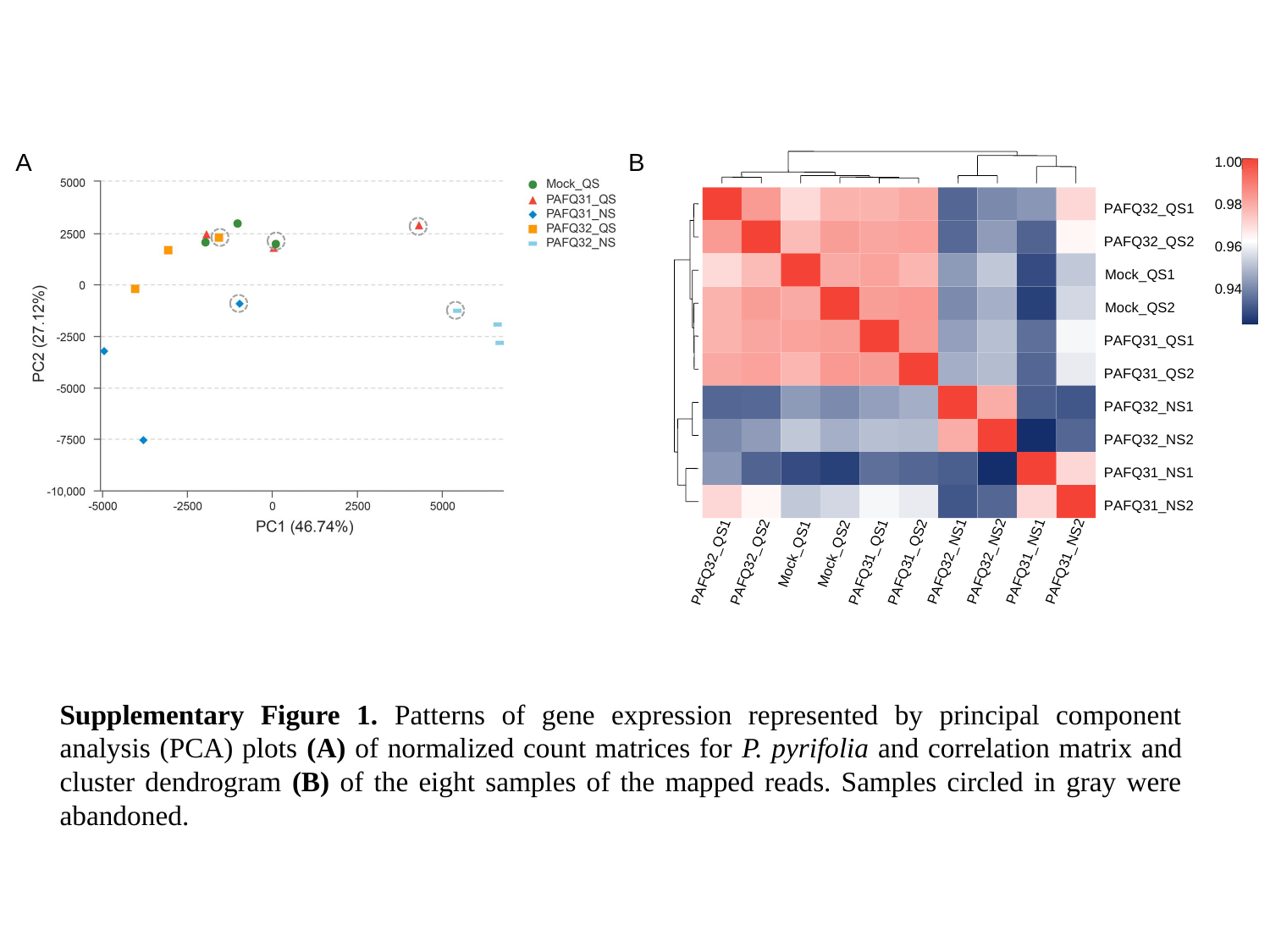

A
B
1.00
0.98
PAFQ32_QS1
PAFQ32_QS2
0.96
Mock_QS1
0.94
Mock_QS2
PAFQ31_QS1
PAFQ31_QS2
PAFQ32_NS1
PAFQ32_NS2
PAFQ31_NS1
PAFQ31_NS2
Mock_QS1
Mock_QS2
PAFQ32_NS1
PAFQ32_NS2
PAFQ31_NS1
PAFQ31_NS2
PAFQ32_QS1
PAFQ32_QS2
PAFQ31_QS1
PAFQ31_QS2
Supplementary Figure 1. Patterns of gene expression represented by principal component analysis (PCA) plots (A) of normalized count matrices for P. pyrifolia and correlation matrix and cluster dendrogram (B) of the eight samples of the mapped reads. Samples circled in gray were abandoned.

## Slide 2
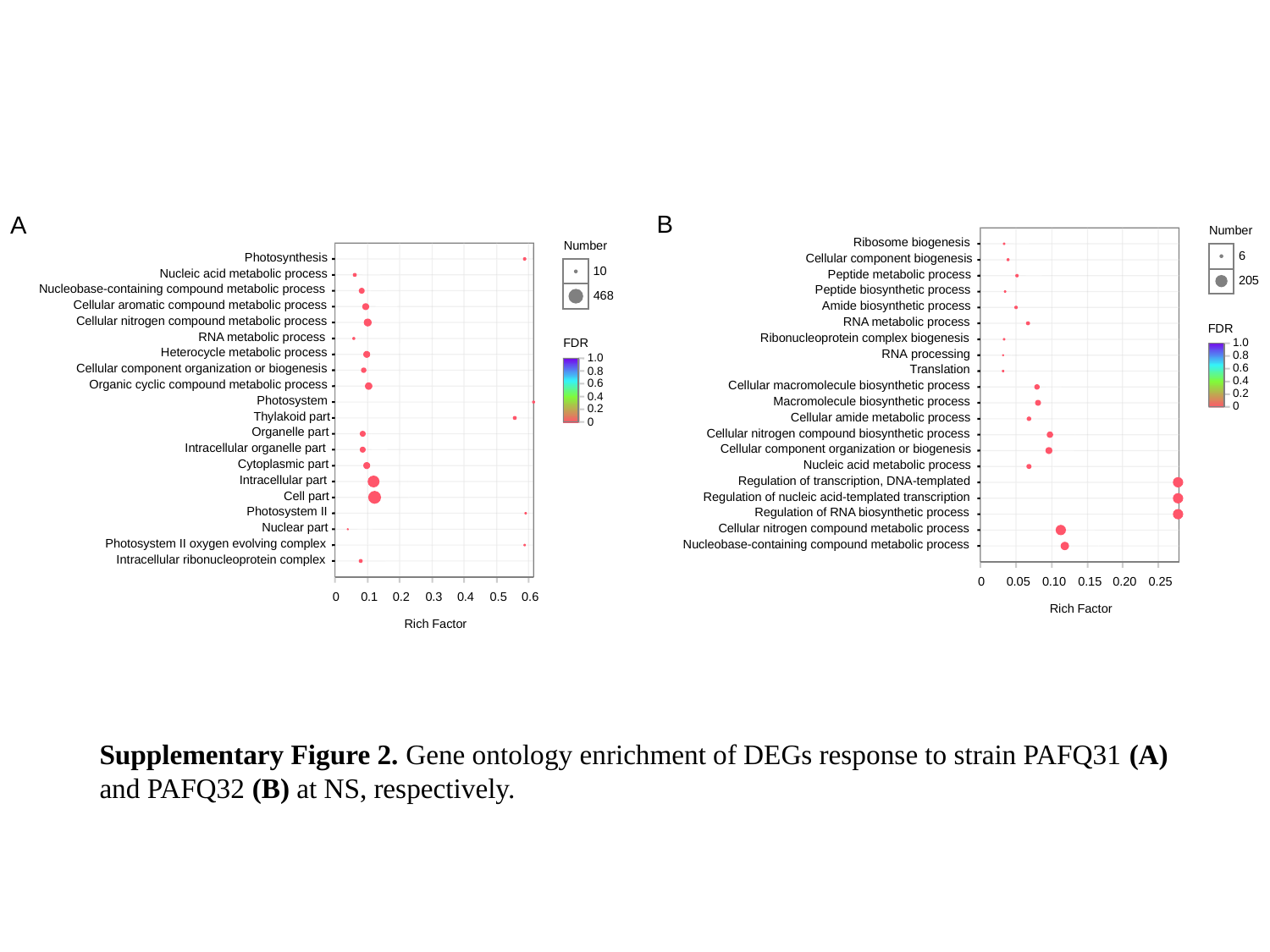

B
Number
Ribosome biogenesis
6
Cellular component biogenesis
Peptide metabolic process
205
Peptide biosynthetic process
Amide biosynthetic process
 RNA metabolic process
FDR
Ribonucleoprotein complex biogenesis
1.0
RNA processing
0.8
0.6
Translation
0.4
Cellular macromolecule biosynthetic process
0.2
Macromolecule biosynthetic process
0
Cellular amide metabolic process
Cellular nitrogen compound biosynthetic process
Cellular component organization or biogenesis
Nucleic acid metabolic process
Regulation of transcription, DNA-templated
Regulation of nucleic acid-templated transcription
 Regulation of RNA biosynthetic process
Cellular nitrogen compound metabolic process
Nucleobase-containing compound metabolic process
0
0.05
0.10
0.15
0.20
0.25
Rich Factor
A
Number
Photosynthesis
10
Nucleic acid metabolic process
Nucleobase-containing compound metabolic process
468
Cellular aromatic compound metabolic process
Cellular nitrogen compound metabolic process
 RNA metabolic process
FDR
Heterocycle metabolic process
1.0
Cellular component organization or biogenesis
0.8
0.6
Organic cyclic compound metabolic process
0.4
Photosystem
0.2
Thylakoid part
0
Organelle part
Intracellular organelle part
Cytoplasmic part
Intracellular part
Cell part
Photosystem II
Nuclear part
Photosystem II oxygen evolving complex
Intracellular ribonucleoprotein complex
0
0.1
0.2
0.3
0.4
0.5
0.6
Rich Factor
Supplementary Figure 2. Gene ontology enrichment of DEGs response to strain PAFQ31 (A) and PAFQ32 (B) at NS, respectively.

## Slide 3
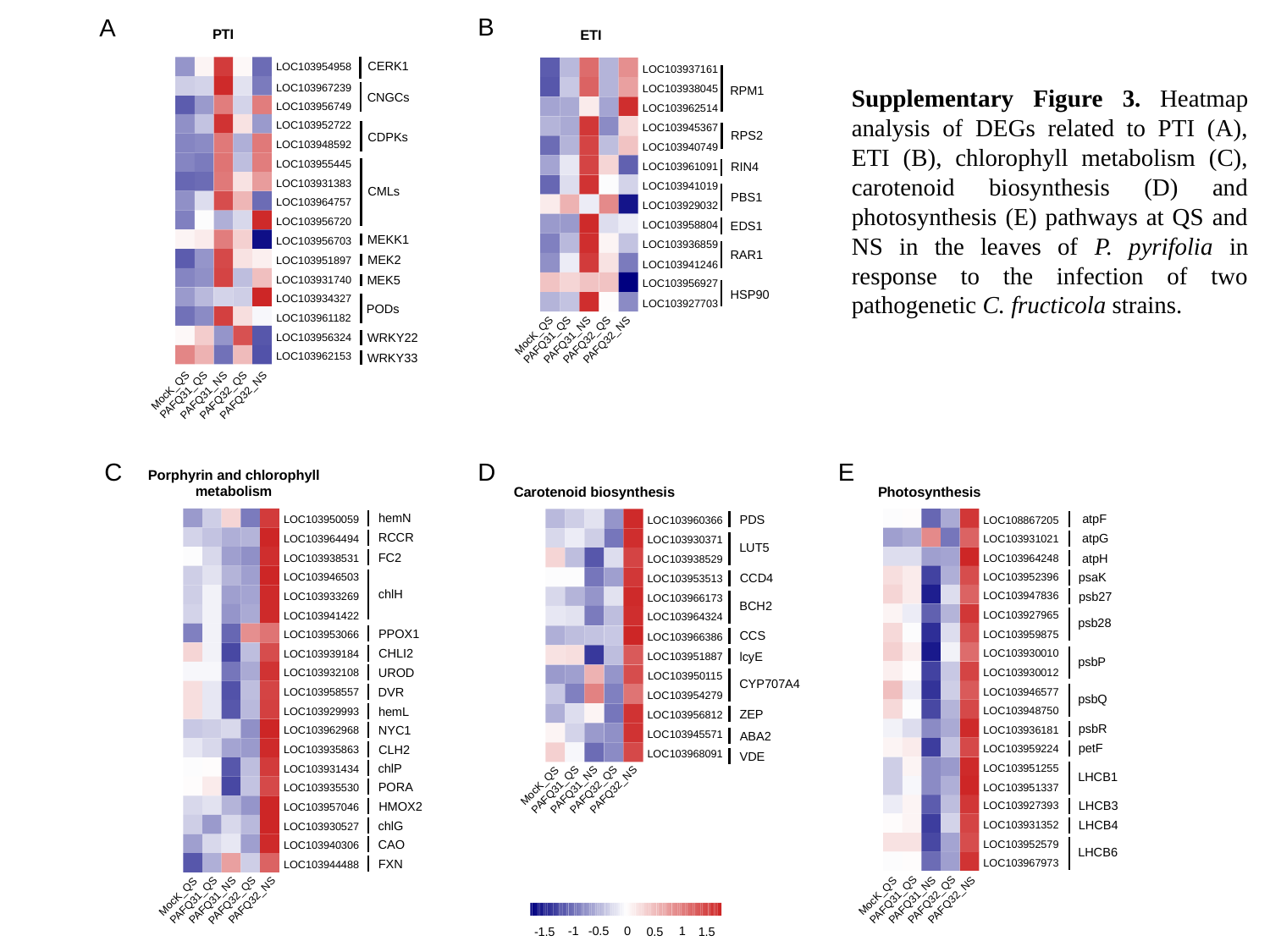

B
ETI
LOC103937161
LOC103938045
LOC103962514
LOC103945367
LOC103940749
LOC103961091
LOC103941019
LOC103929032
LOC103958804
LOC103936859
LOC103941246
LOC103956927
LOC103927703
RPM1
RPS2
RIN4
PBS1
EDS1
RAR1
HSP90
MocK_QS
PAFQ31_QS
PAFQ32_QS
PAFQ31_NS
PAFQ32_NS
A
PTI
CERK1
LOC103954958
LOC103967239
CNGCs
LOC103956749
LOC103952722
CDPKs
LOC103948592
LOC103955445
LOC103931383
CMLs
LOC103964757
LOC103956720
MEKK1
LOC103956703
MEK2
LOC103951897
MEK5
LOC103931740
LOC103934327
PODs
LOC103961182
WRKY22
LOC103956324
LOC103962153
WRKY33
MocK_QS
PAFQ31_QS
PAFQ32_QS
PAFQ31_NS
PAFQ32_NS
Supplementary Figure 3. Heatmap analysis of DEGs related to PTI (A), ETI (B), chlorophyll metabolism (C), carotenoid biosynthesis (D) and photosynthesis (E) pathways at QS and NS in the leaves of P. pyrifolia in response to the infection of two pathogenetic C. fructicola strains.
C
D
E
Porphyrin and chlorophyll metabolism
LOC103950059
LOC103964494
LOC103938531
LOC103946503
LOC103933269
LOC103941422
LOC103953066
LOC103939184
LOC103932108
LOC103958557
LOC103929993
LOC103962968
LOC103935863
LOC103931434
LOC103935530
LOC103957046
LOC103930527
LOC103940306
LOC103944488
hemN
RCCR
FC2
chlH
PPOX1
CHLI2
UROD
DVR
hemL
NYC1
CLH2
chlP
PORA
HMOX2
chlG
CAO
FXN
MocK_QS
PAFQ31_QS
PAFQ32_QS
PAFQ31_NS
PAFQ32_NS
Carotenoid biosynthesis
PDS
LOC103960366
LOC103930371
LUT5
LOC103938529
CCD4
LOC103953513
LOC103966173
BCH2
LOC103964324
CCS
LOC103966386
lcyE
LOC103951887
LOC103950115
CYP707A4
LOC103954279
LOC103956812
LOC103945571
LOC103968091
ZEP
ABA2
VDE
MocK_QS
PAFQ31_QS
PAFQ32_QS
PAFQ31_NS
PAFQ32_NS
Photosynthesis
LOC108867205
 atpF
LOC103931021
 atpG
LOC103964248
 atpH
LOC103952396
psaK
LOC103947836
psb27
LOC103927965
psb28
LOC103959875
LOC103930010
psbP
LOC103930012
LOC103946577
psbQ
LOC103948750
LOC103936181
psbR
LOC103959224
petF
LOC103951255
LHCB1
LOC103951337
LOC103927393
LHCB3
LOC103931352
LHCB4
LOC103952579
LHCB6
LOC103967973
MocK_QS
PAFQ31_QS
PAFQ32_QS
PAFQ31_NS
PAFQ32_NS
1.5
1
0.5
0
-0.5
-1
-1.5

## Slide 4
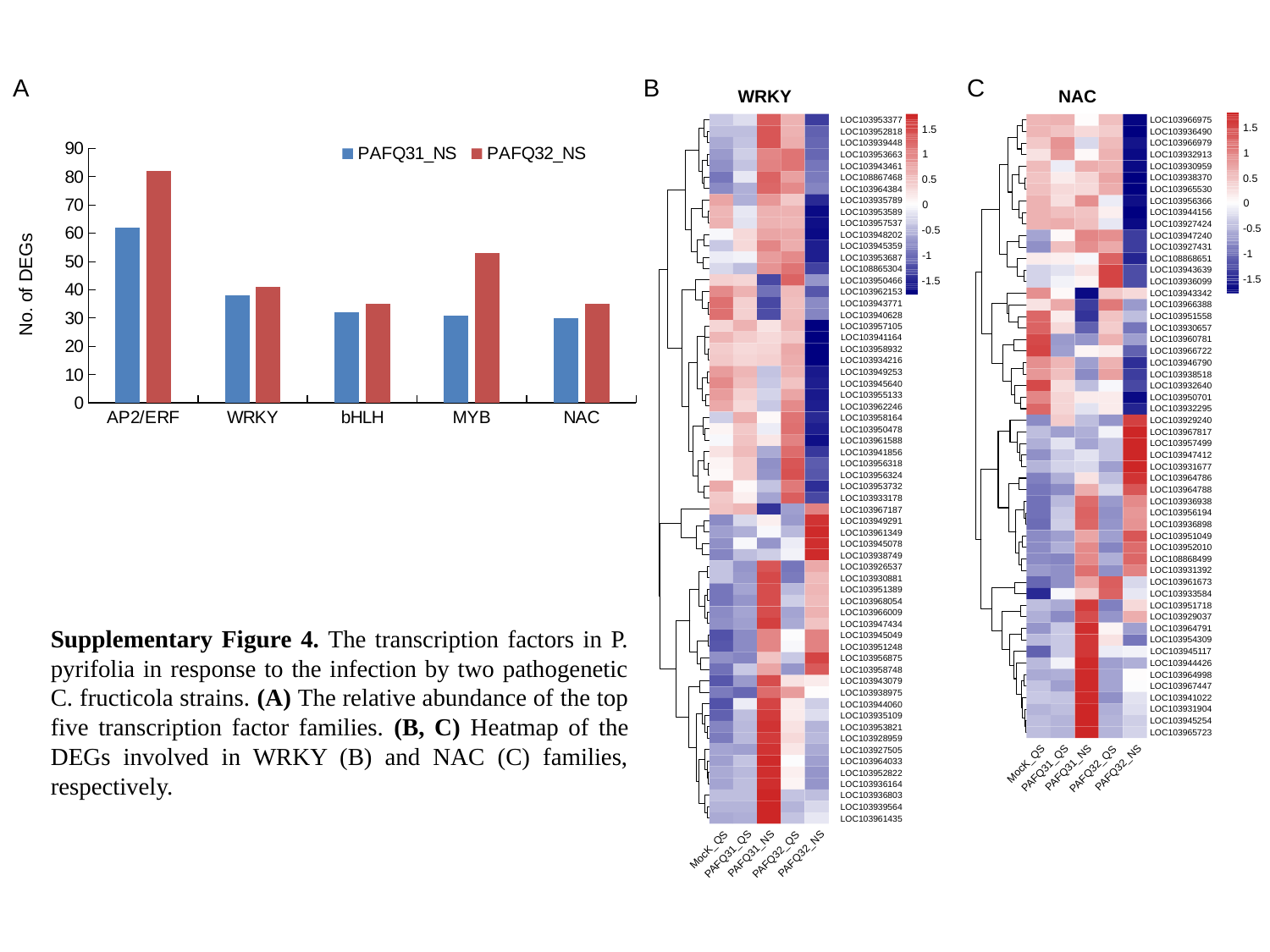

A
B
WRKY
1.5
1
0.5
0
-0.5
-1
-1.5
LOC103953377
LOC103952818
LOC103939448
LOC103953663
LOC103943461
LOC108867468
LOC103964384
LOC103935789
LOC103953589
LOC103957537
LOC103948202
LOC103945359
LOC103953687
LOC108865304
LOC103950466
LOC103962153
LOC103943771
LOC103940628
LOC103957105
LOC103941164
LOC103958932
LOC103934216
LOC103949253
LOC103945640
LOC103955133
LOC103962246
LOC103958164
LOC103950478
LOC103961588
LOC103941856
LOC103956318
LOC103956324
LOC103953732
LOC103933178
LOC103967187
LOC103949291
LOC103961349
LOC103945078
LOC103938749
LOC103926537
LOC103930881
LOC103951389
LOC103968054
LOC103966009
LOC103947434
LOC103945049
LOC103951248
LOC103956875
LOC103958748
LOC103943079
LOC103938975
LOC103944060
LOC103935109
LOC103953821
LOC103928959
LOC103927505
LOC103964033
LOC103952822
LOC103936164
LOC103936803
LOC103939564
LOC103961435
MocK_QS
PAFQ32_NS
PAFQ31_NS
PAFQ31_QS
PAFQ32_QS
C
NAC
1.5
1
0.5
0
-0.5
-1
-1.5
LOC103966975
LOC103936490
LOC103966979
LOC103932913
LOC103930959
LOC103938370
LOC103965530
LOC103956366
LOC103944156
LOC103927424
LOC103947240
LOC103927431
LOC108868651
LOC103943639
LOC103936099
LOC103943342
LOC103966388
LOC103951558
LOC103930657
LOC103960781
LOC103966722
LOC103946790
LOC103938518
LOC103932640
LOC103950701
LOC103932295
LOC103929240
LOC103967817
LOC103957499
LOC103947412
LOC103931677
LOC103964786
LOC103964788
LOC103936938
LOC103956194
LOC103936898
LOC103951049
LOC103952010
LOC108868499
LOC103931392
LOC103961673
LOC103933584
LOC103951718
LOC103929037
LOC103964791
LOC103954309
LOC103945117
LOC103944426
LOC103964998
LOC103967447
LOC103941022
LOC103931904
LOC103945254
LOC103965723
MocK_QS
PAFQ32_NS
PAFQ31_NS
PAFQ31_QS
PAFQ32_QS
No. of DEGs
### Chart
| Category | PAFQ31_NS | PAFQ32_NS |
|---|---|---|
| AP2/ERF | 62.0 | 82.0 |
| WRKY | 38.0 | 41.0 |
| bHLH | 32.0 | 35.0 |
| MYB | 31.0 | 53.0 |
| NAC | 30.0 | 35.0 |Supplementary Figure 4. The transcription factors in P. pyrifolia in response to the infection by two pathogenetic C. fructicola strains. (A) The relative abundance of the top five transcription factor families. (B, C) Heatmap of the DEGs involved in WRKY (B) and NAC (C) families, respectively.
